# The US Deprescribing Research Network: a network to catalyze deprescribing science

**DOI:** 10.3389/fmed.2025.1622200

**Published:** 2025-07-16

**Authors:** Raha Shahroodi, Jonathan D. Norton, Cynthia M. Boyd, Michael A. Steinman

**Affiliations:** ^1^Division of Geriatrics, University of California San Francisco and the San Francisco VA Medical Center, San Francisco, CA, United States; ^2^Division of Geriatric Medicine and Gerontology, Johns Hopkins University School of Medicine, Baltimore, MA, United States

**Keywords:** deprescribing, deprescribe, geriatrics, research network, polypharmacy

## Abstract

Deprescribing plays a critical role in ensuring safe and effective healthcare for older adults, including but not limited to those with vulnerabilities such as multimorbidity and dementia, which increase the risk of harm from unnecessary medications. However, much is unknown on how to safely and effectively stop medications. In response, the US Deprescribing Research Network (USDeN) was created with a mission of enhancing the quality, volume, and real-world impact of research aimed at optimizing medication use among older adults. This Perspective explores the rationale for its creation, the principles and ideas that animate its work, its impacts on research, and future directions for deprescribing research. By advancing research in this area, we aim to improve healthcare outcomes and reduce medication-related harm among older adults.

Deprescribing plays a critical role in ensuring safe and effective healthcare for older adults, including but not limited to those with vulnerabilities such as multimorbidity and dementia, which increase the risk of harm from unnecessary medications (1). However, much is unknown on how to safely and effectively stop medications (2). Recognizing the need to address this evidence gap and grow this field, in 2018 the US National Institute on Aging sponsored a call for the creation of a deprescribing research network. From this, the US Deprescribing Research Network (USDeN) was created with a mission of enhancing the quality, volume, and real-world impact of research aimed at optimizing medication use among older adults. Initially funded under an R24 grant mechanism and later under an R33 grant, USDeN is in its sixth year, continuing many legacy programs while evolving others. With limited budget and effort available, a key theme is to be a force multiplier and maximize impact by finding effective ways to help individuals and groups within the deprescribing research community pursue their own independent and collaborative work to advance the field. In this brief paper, we will discuss some key functions and themes of USDeN and their alignment with needs to advance research on deprescribing.

Investigator development is essential to building the field of deprescribing research. USDeN initially focused on supporting early-career investigators to develop and grow their work in deprescribing, and advance in their career through appointments, promotions, papers, grants that will allow them to continue doing this work in the future. Under the R33, USDeN has expanded its focus to also engage more established investigators by supporting their work in this area, helping them forge connections, skills, and competencies to advance their work.

Broad-based efforts to build community have been an essential component of USDeN’s work. A telling example of this is the USDeN Annual Meeting. These annual meetings attract up to 200 people a year with a focus on sharing new research ideas and methods as well as forging connections and collaborations through small group and interactive sessions. Other activities include monthly newsletters and webinars, where USDeN partners with other deprescribing-focused organizations such as Canadian Medication Appropriateness and Deprescribing Network (CADEN), and other organizations such as the AGING Initiative.

The Junior Investigative Intensive (JII) program was active during the first funding cycle of USDeN. The JII program consisted of work-in-progress meetings, didactics, and community building. This program garnered high interest attracting up to 40 applications per year with 13–15 cohort members selected each year. While successful, when envisioning plans for the next 5 years USDeN leadership felt that it was important to target activities further along the career development pipeline, specifically major grants that will help launch people into productive research careers. As a result, the JII program evolved into the Grant Catalyst Scholars Program. In January 2025, 8 scholars entered the inaugural cohort of the program, which uses a similar format as predecessor program but is targeted to investigators applying for major grants and helping them successfully compete for those grant opportunities.

Another aspect of being a force multiplier are grant opportunities provided by the network. USDeN’s pilot award program supports research related to deprescribing that provides key preliminary data, proof of concept, or developmental work that offers a clear pathway to future, larger-scale studies and career development for the investigators involved. This award is geared primarily, but not exclusively, toward junior investigators. To this end, USDEN has sponsored up to 5 pilot grants each year, selected through a competitive process with a multidisciplinary review panel. As described below, stakeholder engagement is a key theme of the network, and as such plays a critical role in pilot awards. All pilot award proposals are required to provide a stakeholder engagement plan. As part of the selection process, the USDeN Stakeholder Engagement Council reviews pilots alongside scientific reviewers, including evaluations of the quality of the stakeholder engagement activities.

In addition to supporting pilot awardees, USDeN has enjoyed the opportunity to leverage administrative supplements, a unique type of award from the US National Institutes of Health, to further its work. Several of these supplement awards connected to USDeN focused on advancing dementia-related research related to deprescribing, and another supported large pilot studies investigating the use of complementary and integrative health (CIH) modalities to support benzodiazepine deprescribing in older adults.

Principles of stakeholder engagement underpin the research that USDeN supports. The goal of the Stakeholder Engagement Core is to maximize the relevance and impact of research on deprescribing by engaging a range of stakeholders and community partners. A guiding principle is involving stakeholders and community partners both with the research itself and in developing messages to address community concerns and misconceptions about deprescribing. To advance this goal, the network established a Stakeholder Engagement Council to strengthen connections between investigators and stakeholders, both by supporting network-funded research and through stakeholder-facing messaging. As mentioned above, the Stakeholder Engagement Council reviews pilot applications alongside scientific reviewers. This collaborative process led USDeN to develop new methods for integrating stakeholder input into research funding decisions (3).

Once research is funded, awardees present their research while it is process to the Stakeholder Engagement Council for feedback. The Council also provides input on the USDeN website and its content. These and other activities help ensure that our work remains relevant and responsive to the needs of individuals from a wide range of communities and life experiences, so that deprescribing efforts are meaningful and applicable across varied settings.

Another focus of the network has been on developing methodological groundwork that supports a broad variety of future research. There is no single “right” way to do deprescribing research, but building the methodological foundation will provide scientifically and conceptually valid tools and approaches to support a wide range of deprescribing scholarship. Sponsoring a series of projects, entitled “working groups” in USDeN’s first funding cycle and “development projects” in the second, these initiatives are designed to enhance the methodological and conceptual toolkit that will support future research. In the first funding cycle, USDeN sponsored a systematic review of deprescribing studies to identify high-value targets for interventions and associated clinical outcomes, a multisite collaborative project on using electronic health record (EHR) and pharmacy dispensing data to measure deprescribing in different healthcare settings and systems, consensus recommendations on outcomes that are important to measure in deprescribing studies, and the development of an empirically derived conceptual model to advance research and practice on communication (4–7). In the second funding cycle, USDeN is expanding the EHR work to develop measures for medication dose reduction and tapering, as well as strategies for enhancing recruitment and retention of older adults into deprescribing clinical trials. USDeN also developed useful tools to help researchers such as a literature search strategy guidance tool that use broad and narrow search parameters tailored to an investigator’s purpose for the literature search. Finally, USDeN hosts a “Resources for Researchers” page on the USDeN website.^1^

These activities and resources are intended to advance intermediate-term goals while also laying the groundwork for the long-term success and growth of deprescribing science and its translation into routine clinical practice. As deprescribing is a relatively young field, it is critical to pay special attention to supporting promising early-career investigators, giving them the resources, mentorship, opportunities for collaboration, and career development support that will maximize their ability to conduct high-impact deprescribing science, obtain grants, and build their research careers in this area. It is also essential to develop the methodologies, measurement strategies, and other scientific techniques that will serve as the foundation for new science in the years to come. Concurrently, over the long term it is critical to keep eyes on the prize of ensuring that deprescribing science is responsive to the needs of different populations it is intended to serve, and that it be used to effect meaningful change that improves people’s health. These goals pose special challenges, as deprescribing involves the difficult task of changing the behavior of patients, caregiver, and clinicians, and doing so in a health care environment that is not conducive to such changes. No single entity can solve this problem by itself. However, the US Deprescribing Research Network and partner organizations can play a crucial role by promoting attention, resources, and collaborations to support and focus the field’s collective work in addressing these challenging issues.

Deprescribing is a distinct field that requires a broad set of competencies and a truly interdisciplinary approach. Because it directly impacts people, their engagement and buy-in are crucial. This makes stakeholder involvement all the more essential. Through its programs, USDeN has built a research community, developed resources, and catalyzed a wide range of scholarship and research career development, and continues to do so. USDeN’s efforts have yielded numerous successes, including hundreds of papers published and many grants given to its awardees and scholars. But more is still needed. Deprescribing is a growing field and is without a dedicated professional society that calls it home. The US Deprescribing Research Network will continue to serve as a catalyst to the field, helping to advance this important area of research.

## Data Availability

The original contributions presented in the study are included in the article/supplementary material, further inquiries can be directed to the corresponding author.

## Footnotes

^1^ https://deprescribingresearch.org/resources - for - researchers/
